# Draft Genome Sequences of Two Magnetotactic Bacteria, *Magnetospirillum moscoviense* BB-1 and *Magnetospirillum marisnigri* SP-1

**DOI:** 10.1128/genomeA.00814-16

**Published:** 2016-08-11

**Authors:** Veronika V. Koziaeva, Marina V. Dziuba, Timophey M. Ivanov, Boris B. Kuznetsov, Konstantin G. Skryabin, Denis S. Grouzdev

**Affiliations:** Research Center of Biotechnology of the Russian Academy of Sciences, Moscow, Russian Federation

## Abstract

We report here the draft genome sequences of two recently isolated magnetotactic species, *Magnetospirillum moscoviense* BB-1 and *Magnetospirillum marisnigri* SP-1. The genome of *M. moscoviense* BB-1 has 4,164,497 bp, 65.2% G+C content, and comprises 207 contigs. The genome of *M. marisnigri* SP-1 consists of 131 contigs and has a length of 4,619,819 bp and 64.7% G+C content.

## GENOME ANNOUNCEMENT

Magnetotactic bacteria are remarkable for their ability to synthetize magnetosomes, which are prokaryotic organelles representing nanosized magnetic particles enveloped by a belayed lipoprotein membrane. The magnetosome biomineralization is a highly complex process, which is controlled by a set of specific genes lumped together in a single genomic region referred to as magnetosome genomic island (MAI) (1). Magnetosomes are of great interest for their numerous potential biotechnological applications (2–5). In this study, we present the draft genome sequences of *Magnetospirillum moscoviense* BB-1 and *Magnetospirillum marisnigri* SP-1, new magnetotactic strains recently isolated from two rivers in the Russian Federation and described as novel species (6).

Genomic DNA of both species was extracted according to Wilson (7), with minor modifications. The DNA was sonicated on a Covaris S2 device to an average fragment size of 250 bp. Libraries were constructed with NEBNext DNA library prep reagent set for Illumina, according to the protocol for the kit. Libraries were paired-end sequenced in a HiSeq 1500 with 150-bp read length for both reads.

A total of 10,569,046 and 10,022,550 reads providing 100-fold genome coverage were obtained from *M. moscoviense* BB-1 and *M. marisnigri* SP-1, respectively. Genome assembly was performed using SPAdes version 3.1.0 (8). The resulting contigs were submitted to the ProDeGe website (9) for automatic decontamination. Finally, the assembled sequences were automatically annotated using the Prokaryotic Genome Annotation Pipeline (PGAP) provided by the National Center for Biotechnology Information (NCBI) (10).

The draft genome sequence of *M. moscoviense* BB-1 consists of 4,164,497 bp in 207 contigs, with an average G+C content of 65.2%. The genome contained 3,950 genes, of which 3,752 were coding DNA sequences, six coded rRNAs (5S, 16S, and 23S), 46 coded tRNAs, and four were noncoding RNAs (ncRNAs).

The draft genome sequence of *M. marisnigri* SP-1 consists of 4,619,819 bp in 131 contigs, with an average G+C content of 64.7%. The genome contained 4,279 genes, of which 4,130 were coding DNA sequences, six coded rRNAs (5S, 16S, and 23S), 47 coded tRNAs, and four belonged to noncoding RNAs (ncRNAs).

Four gene operons comprising the majority of the genes responsible for magnetosome formation (*mamAB*, *mamDC*, *mms6*, and *mamXY*) were identified in both sequenced genomes. As in other *Magnetospirillum* spp., they are located in a genomic region that has features of a genomic island (11). Genes encoding other common metabolic pathways shown to be involved in magnetosome biomineralization (nitrate reduction, iron transport systems, and ferric reduction) were also found.

### Accession number(s).

The whole-genome shotgun projects of *M. moscoviense* BB-1 and *M. marisnigri* SP-1 have been deposited at DDBJ/ENA/GenBank under the accession numbers LWQU00000000 and LWQT00000000, respectively. The versions described in this paper are versions LWQU01000000 and LWQT01000000.
